# Naturally transmitted mouse viruses highlight the heterogeneity of virus transmission dynamics in the dirty mouse model

**DOI:** 10.1128/jvi.00187-25

**Published:** 2025-05-28

**Authors:** Dira S. Putri, Frances K. Shepherd, Autumn E. Sanders, Shanley N. Roach, Sridevi Jay, Mark J. Pierson, Garritt Wieking, Jodi L. Anderson, David K. Meyerholz, Timothy W. Schacker, Ryan A. Langlois

**Affiliations:** 1 Department of Microbiology and Immunology, University of Minnesota 205104 , Minneapolis, Minnesota, USA; 2 Microbiology, Immunology and Cancer Biology Graduate Program, University of Minnesota 5635, Minneapolis, Minnesota, USA; 3 Department of Lab Medicine and Pathology, University of Minnesota 5635, Minneapolis, Minnesota, USA; 4 Department of Medicine, University of Minnesota 199672 , Minneapolis, Minnesota, USA; 5 Department of Pathology, University of Iowa 160416, Iowa City, Iowa, USA; University of Michigan Medical School, Ann Arbor, Michigan, USA

**Keywords:** dirty mice, natural mouse viruses, virus tropism, virus transmission

## Abstract

Specific-pathogen-free (SPF) mice are widely used in biomedical research to model human infections. However, these animals do not always accurately recapitulate human immune responses. This is due, in part, to their lack of infection history. A growing number of studies show that the host microbiome influences the development, progression, and responses of many diseases. To date, the majority of research on the microbiome has focused on the bacterial populations and less on the eukaryotic virome of the host. Here, we characterize a transmission model where SPF mice are exposed to natural mouse pathogens at physiologic doses and routes. We found that pet store mice acquired from different sources have distinct viromes and infection histories. We also found significant heterogeneity in the kinetics of the transmission of natural mouse viruses. A common virus found in our model was murine Kobuvirus. Surprisingly, murine Kobuvirus infection was found in the glandular stomach epithelia and not intestinal epithelia like other enteric picornaviruses. Together, these data characterize the heterogeneity of the dirty mouse cohousing system and provide a foundation for studying the biology of natural mouse viruses.

**IMPORTANCE:**

Increasing evidence supports microbial exposure as a critical factor in shaping responses to immune challenges such as infections and vaccinations. However, many experimental models introducing microbial exposure into laboratory animals have confounding factors that may impact phenotypes and are not well characterized. Here, we characterized the pet store reservoir virome diversity, prior infection history, and transmission kinetics. We found significant heterogeneity across these features of the pet store cohousing model. Moreover, we leveraged this model to investigate the tropism of two less characterized viruses—murine Kobuvirus and murine astrovirus 2—in a natural transmission setting. These findings highlight the importance of characterizing the virome of pet store reservoirs to better mimic microbial exposure in humans.

## INTRODUCTION

Traditional animal models of infections use laboratory-grown human viruses in an attempt to model human infections. Termed reverse zoonosis, these experimental cross-species infections often require manipulation of the virus or the animal host and do not always capture the natural pathology, transmission, and immune responses seen in humans. They also may miss other features experienced in real-world environments, including co-infections, circulating virus diversity, natural exposure routes, and infectious doses. This is particularly true for transmission studies. The bacterial microbiome has garnered extensive attention because of its association with many adverse health outcomes. In contrast, few studies focused on the virome component of the host-associated microbiome (1–3) partly due to the lack of a universal conserved gene (such as the 16S rRNA gene in bacteria) which hinders the identification and classification of different viruses. To address these shortcomings, we developed a natural transmission model where mice from the pet store, which have a diverse pathobiome, serve as a virus reservoir and are cohoused with specific-pathogen-free (SPF) laboratory mice (4, 5). This system results in the transmission of diverse microbes through natural transmission routes and doses in a controlled laboratory setting.

The pet store cohousing system has also been used to provide genetically tractable laboratory mice with a complex infection history to better mimic human immune status and improve preclinical models of human diseases (6). Preclinical therapeutic findings done in SPF mice have been shown to translate poorly to humans. For example, prophylactic administration of anti-tumor necrosis factor monoclonal antibody was able to protect SPF mice from lethal endotoxemia but failed to rescue in a model with natural microbial exposure (7, 8). These differences have been attributed to the impaired immune maturation of SPF mice due to their limited microbial exposure. Various studies have shown that, compared to SPF mice, pathogen-experienced mice exhibit vaccine responses against influenza (9), severe acute respiratory syndrome coronavirus 2 (10), and yellow fever (11) that better recapitulated those observed in humans. We also recently determined that the degree of pathogen exposure can also impact subsequent responses to vaccination (12). Additionally, dirty mice also better model phenotypes of human immunodeficiency than SPF mice (13). These studies demonstrate that microbial exposure—particularly viruses—can provide essential cues impacting the development of the immune system, disease manifestation, and responses to other infections. Furthermore, these data highlight the power of this model system and emphasize the need to better characterize the heterogeneity and the confounding factors that may affect the response to pathogen exposure. These outstanding questions include the duration of exposure, dose at the time of exposure, the number of sequential infections, and the effect of immune responses induced by prior chronic and acute infections.

The pet store cohousing model enables us to characterize virus and host features that are necessary to evaluate virus transmission. These “dirty” pet store mice harbor many rodent pathogens that reflect the natural biological diversity of viruses, making them excellent reservoir hosts. The model provides access to the entire transmission chain, starting from the pet store mouse reservoir to the new host. This makes serological and nucleic acid-based pathogen surveillance of both the new hosts and reservoirs achievable. In this study, we characterized the dynamics of virus transmission in the natural pet store cohousing transmission model. We conducted comparative assessments of different dirty mouse colonies that carry distinct viruses and have unique infection histories. We found significant heterogeneity in the shedding kinetics of natural mouse viruses from the pet store mice upon arrival to our laboratory.

We further interrogated murine astrovirus 2 (MuAstV2) and murine Kobuvirus (MKV), which were the most prevalent viruses in our model system. MuAstV2 was initially found in a laboratory mouse colony and is genetically distinct from MuAstV1 (14). Despite its prevalence in wild and laboratory mouse populations, its pathogenesis remained largely understudied. Conversely, MKV is a much less characterized enteric virus. Kobuviruses are single-stranded RNA viruses in the family *Picornaviridae*. Their genome comprises a leader (L) protein encoded at the N-terminus followed by three structural capsid proteins (VP0, VP3, and VP1) and seven non-structural proteins (2A–2C and 3A–3D). Kobuviruses are distributed worldwide and associated with gastroenteritis not only in humans but also in a number of domestic and wild animals (15). To date, these viruses have been found in various host orders, including Primate, Lagomorpha (16), Chiroptera (17, 18), Artiodactyla (19, 20), Carnivora (21), Aves (22, 23), and Rodentia (24). Kobuviruses are currently separated into six species, Aichivirus A to F. Human and rodent kobuviruses belong to Aichivirus A species, and they share >70% amino acid sequence identity in the capsid protein region and >80% amino acid sequence identity in the 2C and 3CD regions (25). Although kobuviruses constitute a significant public health concern, particularly in immunocompromised individuals (26, 27), there are no treatments or vaccines available due to the limited knowledge about the pathogenesis and mechanisms by which these viruses cause disease. To understand its pathogenesis and fundamental viral immunity in immunocompromised patients, a small animal model needs to be established. We propose that further characterization of MKV would be useful to serve as a model for human Kobuvirus infections and pathogenesis.

In summary, we found significant heterogeneity in stages of infection, levels of virus shedding, and transmission kinetics in the natural mouse model of transmission. Our highly tractable model system offers the ability to conduct comparative assessments of infection histories for pet store mice derived from different sources. With our model, we identified cellular tropisms of several understudied natural mouse viruses that could be further characterized as infection models for related human virus infection studies.

## RESULTS

### Heterogeneous stage of infection and virus shedding upon pet store mouse arrival to the laboratory

**Fig 1:**
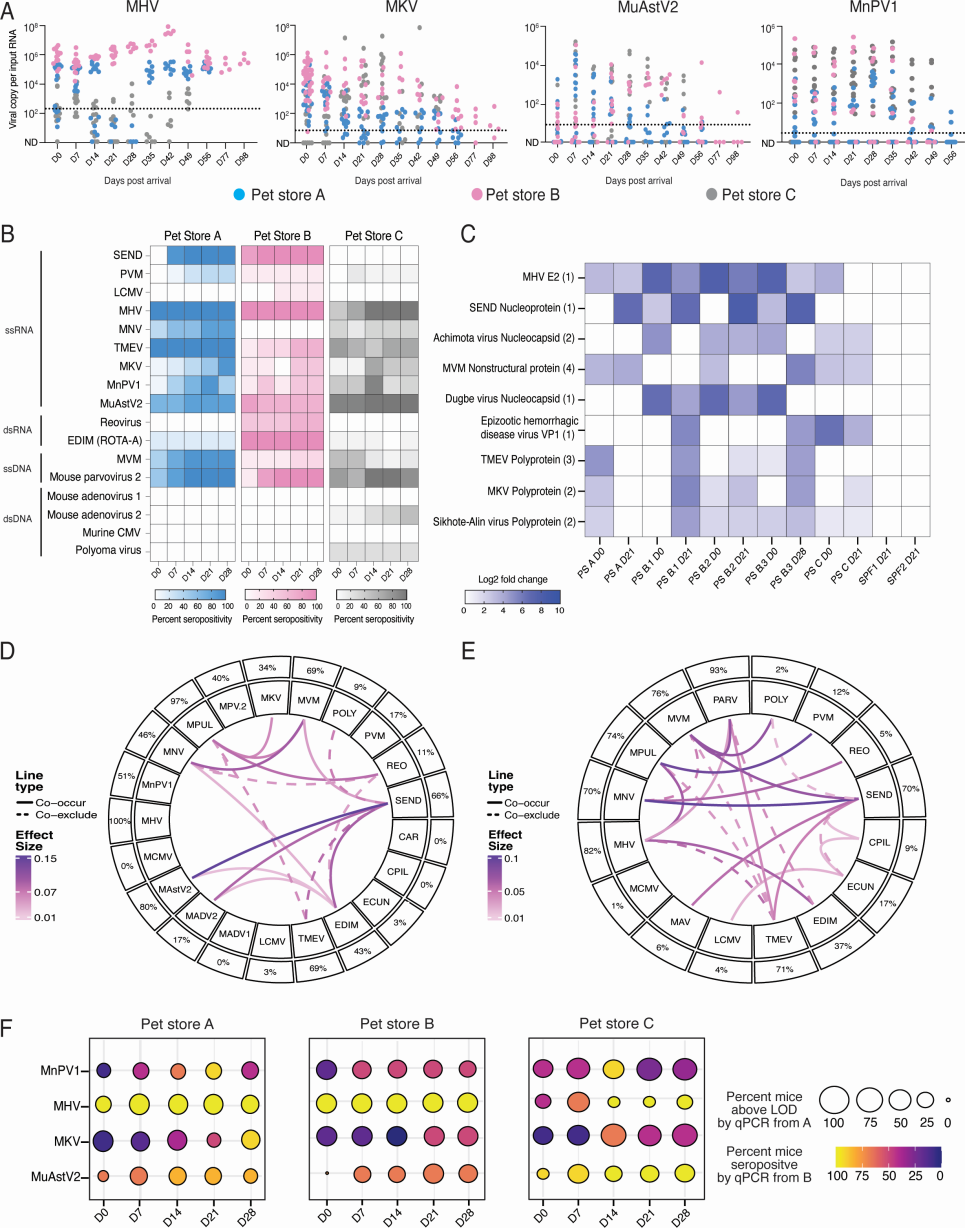
Characterization of pet store mice viral shedding dynamics and infection history. (**A**) qRT-PCR analysis of viral load in shed fecal pellets across time. Each dot represents one mouse, and color indicates the pet store of origin. Dotted lines indicate the limit of detection (LOD) based on SPF background. (**B**) Serology analysis of pet store mouse infection history from the day of arrival (day 0 [D0]) until the time of sacrifice (day 28 [D28]). (**C**) Select pet store mice from three different pet stores profiled for their infection history at D0 and D28 using the VirScan platform. Three pet store mice were selected from pet store B, denoted as B.1, B.2, and B.3. Numbers inside parentheses represent the number of peptides mapped to the virus. PS, pet store. (**D**) Co-occurrence analysis of all screened pet store mice at D28 from panel A (*n* = 35 mice). (**E**) Co-occurrence analysis of data from panel **D** combined with historical pet store mice serology data at D60 (*n* = 164 mice). (**F**) Dot plot comparing frequencies of seropositivity and fecal viral load above LOD by reverse transcription quantitative PCR. The percentage of pet store mice with fecal viral loads above the LOD (dot size) and their percent seropositivity (dot coloration) were calculated. (**A **and **B**) Pet store A, *n* = 14 mice; pet store B, *n* = 6 mice; pet store C, *n* = 15 mice. Abbreviations for pathogens in the serology panel can be found in Materials and Methods.

We sought to identify the viral shedding dynamics and infection stages of the naturally occurring viruses in pet store mice by evaluating fresh fecal pellets and sera over time from mice sourced from three different pet stores. On the day pet store mice arrived at our housing facility (day 0), RNA from fresh fecal pellets was analyzed by quantitative reverse transcription PCR (qRT-PCR) for murine hepatitis virus (MHV), murine astrovirus 2 (MuAstV2), murine Kobuvirus (MKV), and Minnesota rodent picornavirus 1 (MnPV1), a virus which we recently discovered in pet store mice (5). We observed distinct shedding dynamics of different viruses and between animals from different pet stores (Fig. 1A). For example, mice from pet store B persistently shed MHV at a higher load compared to mice from pet stores A and C. In contrast, mice shed MuAstV2 and MnPV1 at comparable levels regardless of pet store origin. Pet store mice shedding MuAstV2 started to clear the infection at around 49 days post-arrival (dpa), although one mouse from pet store B was shown to have detectable levels of MuAstV2 in the feces through 98 dpa. Forty-seven percent of pet store mice ceased shedding MuAstV2 in the feces by day 28, whereas 16.7% and 23.3% of pet store mice had no detectable levels of MKV and MnPV1, respectively, at that time point. Interestingly, 93.75% of pet store mice were still shedding MHV at day 49, suggesting that MHV infection persisted longer than MuAstV2, MKV, and MnPV1 in the pet store mice. These data demonstrate the heterogeneity of viral shedding dynamics in this model system.

To determine if animals were in an early acute stage of infection upon acquisition, we evaluated the serology status of pet store mice weekly after arrival using an SPF serology panel. We also developed peptide-based enzyme-linked immunosorbent assays (ELISAs) for three of the most common viruses in pet store mice not included in traditional SPF panels: MuAstV2, MKV, and MnPV1. We validated these in-house peptide ELISAs for specificity and sensitivity (Fig. S1). Serological testing of pet store mice revealed that many animals were positive for commonly found viruses in the pet store mice; however, we also observed seroconversion as early as 7 dpa. Pet store mice that were shedding MHV were seropositive upon arrival, suggesting that mice chronically infected with MHV were also captured in this study (Fig. 1B). Together, these data suggest that we are also capturing some mice that are in the acute stage of infection with natural mouse viruses.

Previous serological studies of pet store and wild mice have relied primarily on targeted ELISAs and bead-based immunofluorescence assays for serosurveillance. However, screening by these techniques is constrained in the breadth of potential targets that can be simultaneously assayed, thus limiting their utility in the surveillance and discovery settings. To address this, we utilized VirScan, a high-throughput method to comprehensively analyze antiviral antibodies using phage immunoprecipitation and DNA sequencing of the phage library displaying proteome-wide peptides of all known vertebrate viruses (28). We used this approach to ensure that we captured the infection histories by all known viruses and limit biases generated from mouse pathogen-specific serology panels. Sera from representative pet store mice sourced from three different pet stores were profiled using the VirScan library on a PhIP-Seq platform. VirScan detected antibodies capable of binding to an array of both DNA and RNA viruses in pet store mice (Fig. S2). However, the platform also detected potential non-specific hits such as viruses that are not known to infect mice, insect viruses, and plant viruses. As expected, viruses identified by the SPF panel and our ELISA were also captured in our VirScan analysis, including Sendai virus (SEND), MHV, Theiler’s murine encephalomyelitis virus (TMEV) of the GDVII strain, and MKV (Fig. 1C). We also found hits for viruses that are not included in traditional mouse serological panels but are known to infect mice, such as Achimota virus (*Paramyxoviridae*), Dugbe virus (*Bunyaviridae*), and Sikhote-Alin virus (*Picornaviridae*). Together with multiplex fluorometric immunoassay panels and our in-house peptide ELISA, VirScan provided a cohesive picture of the pet store mice virome, which included viruses rarely detected in the commercially available serological panels.

A highly heterogeneous system allowed us to explore the relationship between viruses in the infection history of pet store mice. We performed co-occurrence analysis in which we measured how frequently a pet store mouse is seropositive for two pathogen species at the same time (29). Within all the store mice screened at day 28 (Fig. 1A), we saw the strongest co-occurrence between MuAstV2 and SEND and the strongest co-exclusion effect between SEND and TMEV (Fig. 1D). When we combined all of the screened pet store mice (Fig. 1A) with historical serology data from pet store mice profiled at day 60 (9), we saw that the strongest co-occurrence was observed between murine norovirus (MNV) and SEND and between *Mycoplasma pulmonis* (MPUL) and pneumonia virus of mice (PVM). Pet store mice seropositive for TMEV were shown to be less likely to be seropositive for MHV, MPUL, and minute virus of mice (MVM) (Fig. 1E). In both the current cohort and combined historical serology data, TMEV was found to be the primary determinant for co-exclusion with other viruses. We next evaluated the relationship between shedding viral load and serostatus in pet store mice over time. Pet store mice with detectable MHV viral loads in the feces (dot size) are also seropositive for MHV (dot coloration) at the day of arrival, suggesting that these mice are chronically infected with MHV upon acquisition to our facility. In contrast, pet store mice shedding MuAstV2 at day 0 become seropositive over time starting at day 14 post-cohousing, indicating that these mice were in the acute phase of infection upon acquisition (Fig. 1F).

Together, these data describe that pet store mice exhibit diverse stages of infection, heterogeneous virus shedding, and complex co-infections, which can be used as a tractable model to study virus transmission biology.

### Rapid and heterogeneous virus transmission during cohousing

**Fig 2:**
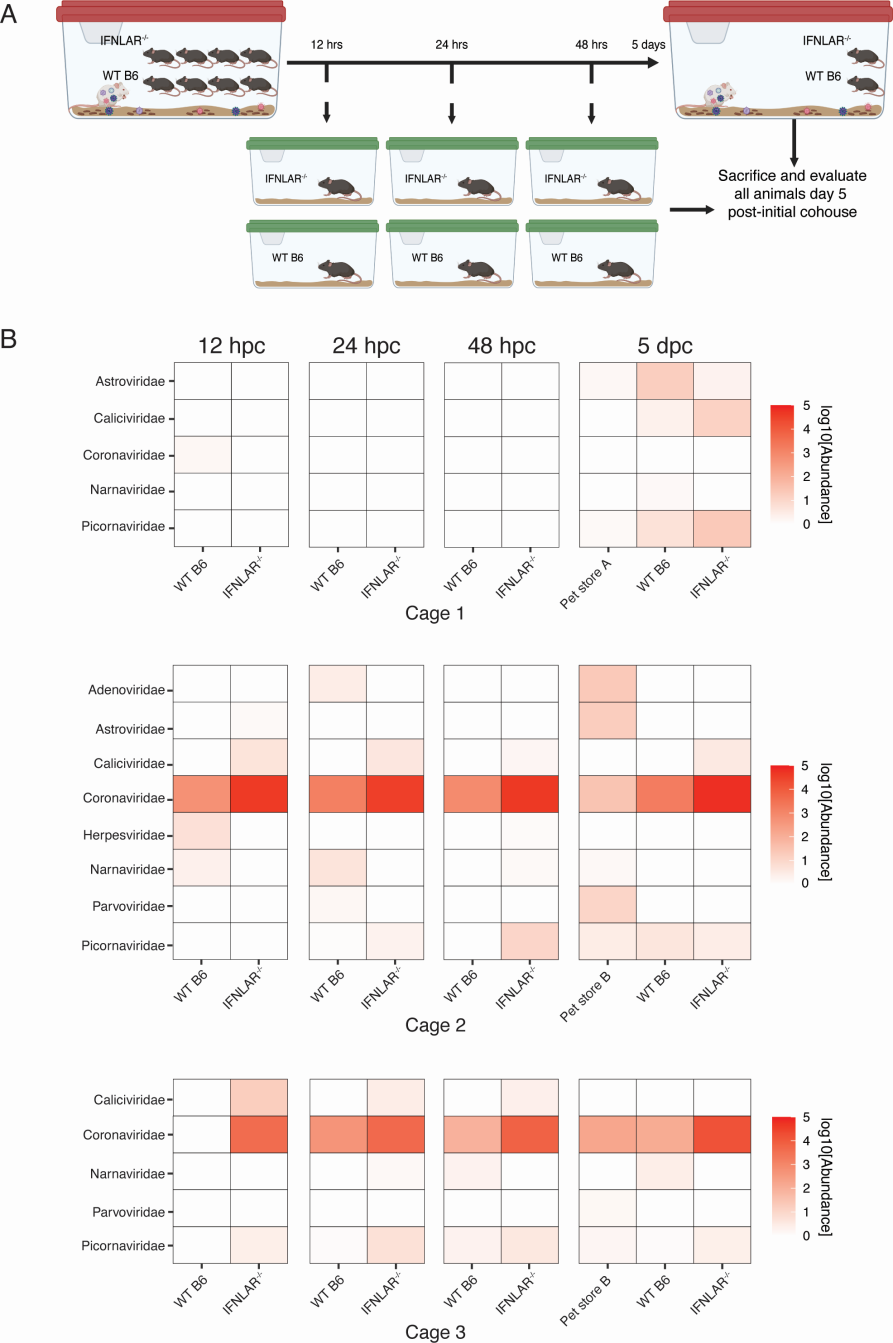
Rapid and heterogeneous transmission of natural mouse viruses to cohoused mice. (**A**) Model of time course transmission experimental design. (**B**) Normalized read counts of virus families detected in the small intestine by bulk RNAseq. Data are from three independent cages of pet store mice experiments. Mice from pet store A were used for the cohousing setup of cage 1, while mice from pet store B were used for the cohousing setup of cages 2 and 3. dpc, days post-cohousing; hpc, hours post-cohousing.

Thus far, we have shown that pet store mice shed different viruses at different rates, and each of them possesses a distinct infection history. Next, we evaluated the transmission kinetics of these natural mouse viruses. Pet store, SPF wild-type (WT) C57BL/6 (B6), and laboratory mice lacking interferon (IFN)-alpha and IFN-lambda receptors (IFNLAR^−/−^) were cohoused together for either 12, 24, and 48 hours or 5 days. At each time point, one set of B6 and IFNLAR^−/−^ mice was removed from the parent cage and individually housed until day 5 post-initial exposure (Fig. 2A). To identify viruses that were transmitted from pet store mice to the cohoused mice, we sequenced polyA-selected RNA from the pet store reservoir and the cohoused SPF hosts. We assessed the small intestine as one site of replication for viruses transmitted through the fecal-oral route. We performed three independent experiments with independent mice acquired from pet stores A and B, which had unique starting viromes. We observed viruses in the family *Coronaviridae* to be rapidly transmitted as early as 12 hours post-cohousing (Fig. 2B). Given the heterogeneity inherent to the pet store mouse cohousing model, we also observed different rates in which other viral families transmit to the SPF hosts. For example, in two experiments, *Caliciviridae* rapidly transmitted only in the absence of IFN signaling. Additionally, in some instances, viruses in the *Astroviridae* family took several days of exposure for detectable transmission. This time course transmission data highlight the diversity and kinetics of virus families that can be transmitted to the cohoused mice in our dirty mouse model.

### Cellular infection landscape of the pet store and SPF mice large and small intestine epithelium

**Fig 3:**
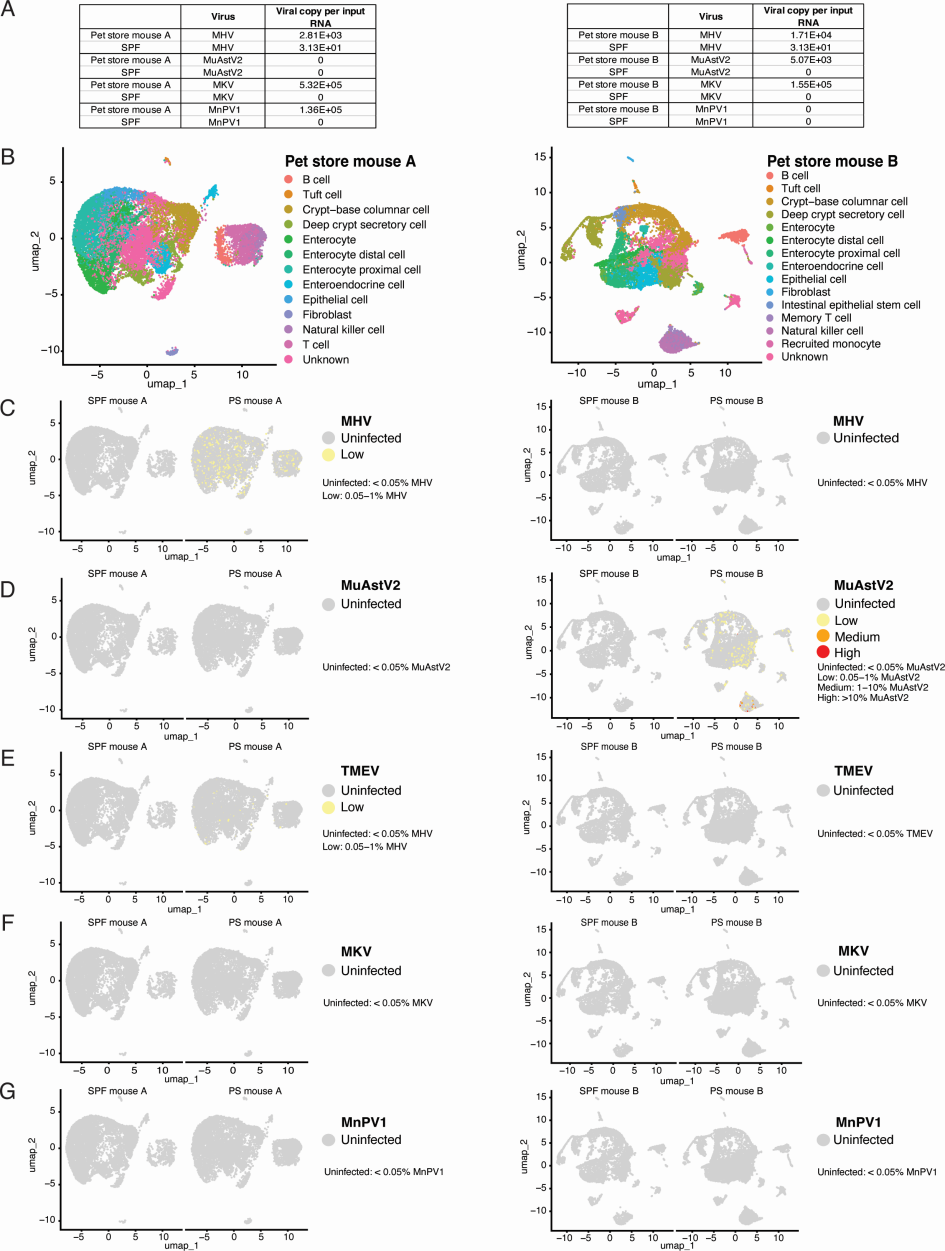
Single-cell transcriptomics of pet store mouse small and large intestines. (**A**) qRT-PCR viral load in shed fecal pellets at D0 for pet store mouse A and pet store mouse B. (B, left) scRNAseq cell clusters integrated Uniform Manifold Approximation and Projection (UMAP) of pet store mouse A and SPF mouse A colored by cell types assigned with ScType. A total of 8,924 and 7,639 cells were analyzed for pet store mouse A and SPF mouse A, respectively. (B, right) scRNAseq cell clusters integrated UMAP of pet store mouse B and SPF mouse B. A total of 9,368 and 6,455 cells were analyzed for pet store mouse B and SPF mouse B, respectively. Feature plots of percent (**C**) MHV, (**D**) MuAstV2, (**E**) TMEV, (**F**) MKV, and (**G**) MnPV1 gene expression per cell in the integrated data sets.

The pathogenesis of many viruses found in these mice, including MuAstV2 and MKV, is vastly understudied. Additionally, the cellular tropism of MKV is unknown. To determine the cellular tropism of natural mouse viruses in the digestive tract, we profiled the large and small intestinal epithelia by single-cell RNAseq. We screened two pet store mouse feces for MKV, MHV, MnPV1, and MuAstV2. We observed shedding of each of these viruses across the two pet store mice (Fig. 3A). To probe which epithelial cells were infected, we enriched for intestinal crypts to ensure the capture of all epithelial cell lineages along the crypt-villus axis (30). After removing low-quality cells (see Materials and Methods), we determined the clustering resolution using clustree, performed unsupervised clustering, and identified 13 (pet store mouse A) and 15 (pet store mouse B) distinct clusters based on a combination of established markers and ScType cell annotation (Fig. 3B). Of note, immune cells (T cells, B cells, and natural killer [NK] cells) are common “contaminants” in epithelial preps due to their abundance in the tissue. We found MHV-infected cells in multiple cell type clusters in pet store mouse A, especially across different enterocytes (Fig. 3C, left). In pet store mouse B, we found cell clusters positive for MuAstV2 (Fig. 3D, right), particularly in the enterocyte clusters and in NK cells. This finding is consistent with a recent study where MuAstV2 RNA hybridization was found to co-localize with enterocyte (cytokeratin) markers (31). We detected cells infected with TMEV at a low level in pet store mouse A (Fig. 3E, left), although no TMEV-infected cells were found in pet store mouse B (Fig. 3E, right). Surprisingly, we could not find any reads for MKV and MnPV1 in both pet store mice (Fig. 3F and G), despite detecting virus shedding in the shed feces by qRT-PCR (Fig. 3A). This led us to explore other tissues in the gastrointestinal tract to pinpoint the tissue and cellular tropism of MKV.

### The glandular stomach is the main site of MKV infection

**Fig 4:**
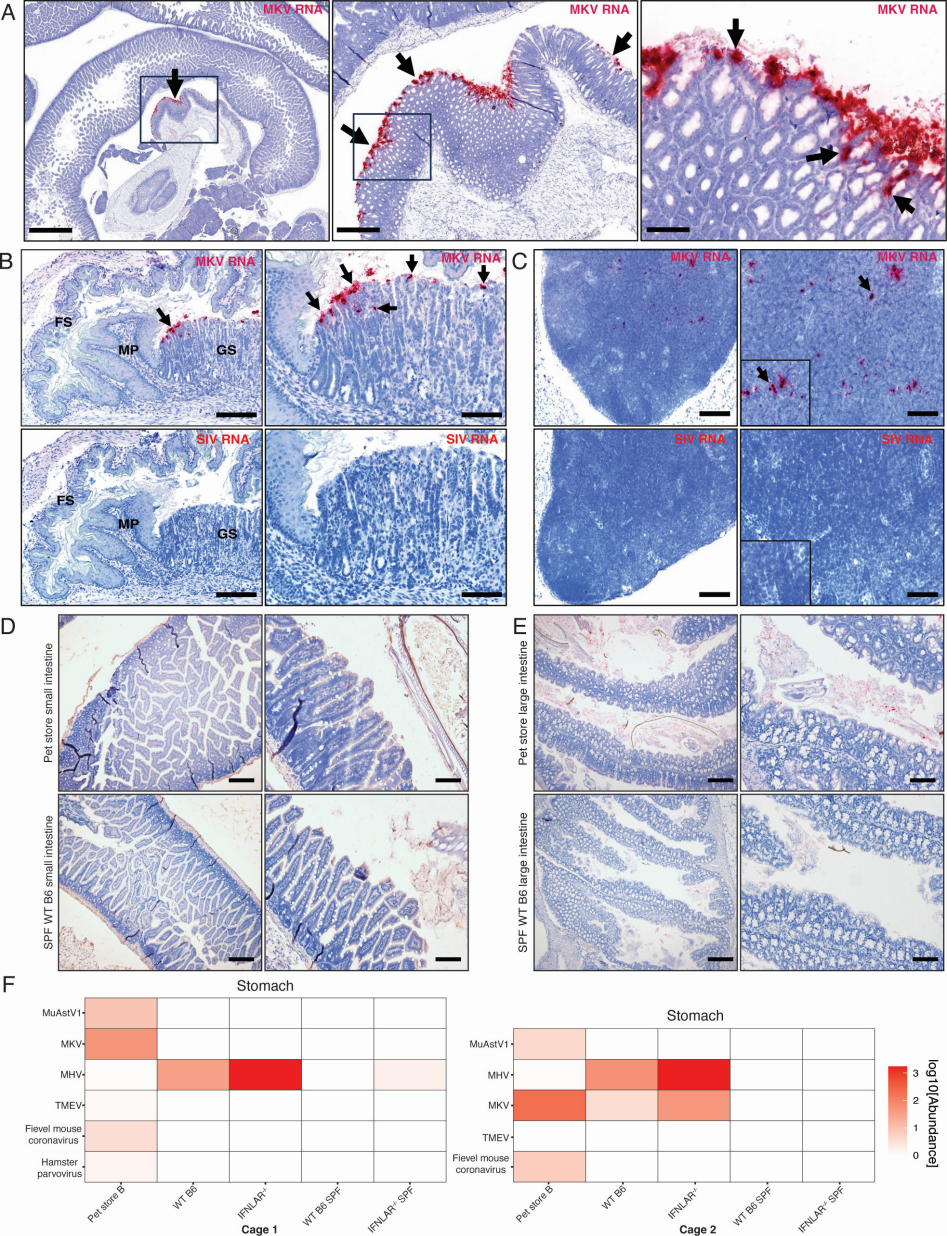
MKV localization in the infected pet store mouse. (**A**) Swiss roll of small intestine with antral stomach from a pet store mouse. RNAscope probe for MKV (red denotes MKV RNA) showed coalescing to multifocal staining (arrows) of surface epithelium in the antral to pyloric regions of the glandular stomach, but staining was absent from intestines. Scale bar = 775, 163, 35 µm, respectively. (**B**) Sections of stomach at the level of the margo plicatus (MP) in pet store mice were probed for MKV (top) or SIV (bottom). RNAscope probes showed coalescing to multifocal staining (arrows) of the surface epithelium in the glandular stomach (GS), but an absence of staining was observed in the adjacent forestomach (FS) and in all the SIV probed tissues. Scale bar = 176 µm. (**C**) Sections of gastric lymph node from pet store mice probed for MKV (top) and SIV (bottom). Arrows and inset denote scattered MKV staining. Scale bar = 188 and 94 µm, respectively. (D and E) Intestines from pet store (top) or SPF (bottom) mice. Small (**D**) and large (**E**) intestinal tissues were probed for MKV, but all lacked staining. Scale bar = 200 and 100 µm, respectively. (**F**) Normalized read counts of virus families detected in the stomach by bulk RNAseq (log10 transcripts per million [TPM]) after 72 hours of cohousing. Data from two independent pet store mice transmission experiments.

To determine the cellular and tissue tropism of MKV, we probed for the location of MKV replication using *in situ* hybridization (ISH) and RNAscope. For this study, we prescreened for pet store mice with a highly detectable MKV viral load in the feces upon arrival and harvested the stomach, small intestine, and large intestine the next day. Tissues were probed against MKV whole genome or simian immunodeficiency virus (SIV) as a negative control. In the antral to pyloric regions of the glandular stomach, strong MKV staining was observed in coalescing to multifocal surface epithelia with some extension into the gastric pits (Fig. 4A). Further evaluation of the stomach at the interface of the glandular stomach and forestomach showed similar staining patterns in the glandular stomach, but staining was absent in the forestomach (Fig. 4B). We also identified multifocal MKV staining in the gastric lymph nodes but not in any mesenteric lymph node (Fig. 4C and data not shown, respectively). No staining for MKV was observed in the cells of either the small or large intestine (Fig. 4D and E). When MKV-positive pet store mice were cohoused with WT B6 and IFNLAR^−/−^ mice for 72 hours, we were able to detect MKV in the stomach of some mice by RNA sequencing (Fig. 4F). Together with the *in situ* hybridization data, we suggest that MKV replicates in the glandular stomach but can potentially be identified in the distal digestive tract as it is being shed in the feces.

## DISCUSSION

Experimental animal models that incorporate infection history and immune experience are helping to improve the study of immune responses and the biology of virus transmission. Here, we characterized the viral heterogeneity of the dirty mouse model system. We found that various stages of viral infection were represented in the pet store mice, and they exhibit heterogeneous shedding kinetics across virus species. Pet store mice from three different sources were evaluated for their viral infection dynamics. We saw seroconversion of many commonly found viruses in the pet store mice by 7 dpa; however, we also observed that mice shedding high levels of MHV were seropositive on the day of arrival. This suggested that we captured both acutely and chronically infected animals. We observed both co-occurrence and co-exclusion relationships between multiple viruses in cohoused mice. In particular, we found the strongest co-exclusion effect against SEND, MHV, MPUL, and MVM in mice seropositive for TMEV. This is consistent with previous cohousing studies in which we observed co-exclusion of *Picornaviridae* and *Coronaviridae* during transmission (4). There is a growing body of literature on the impacts of the virome on health and disease. These data help reveal complex interactions within the virome and offer a model to investigate the mechanism of co-occurrence and co-exclusion between different viruses.

We also evaluated MKV virus tropism and suggest this virus as a potential mouse model for Aichivirus infections in humans.

Virus shedding for acute infections is transient, thus limiting insights gained from nucleic acid testing of fecal pellets from pet store mice that have been housed for a long period of time. Additionally, SPF mouse serology panels only include the most commonly found viruses in laboratory mice. Using VirScan, we circumvented these caveats and identified past exposures to viruses that are not detectable using available commercial serology panels. For example, the Sikhote-Alin virus was never detected in our sequencing approach as well, suggesting that there might be cross-reactivity between these viruses and other viruses from the same families, particularly in the *Picornaviridae* family. Together, our data demonstrate the utility of using large-scale phage display serology assays for longitudinal viral surveillance efforts to better understand the infection profile of pet store mice used as models of virus transmission and normalized infection history for immunology.

Intestinal homeostasis and the composition of epithelial cells can be impacted by the host’s microbiota in a community and species-dependent manner (32–34). However, there is a lack of understanding of how the host’s virome impacts intestinal homeostasis. In a first step toward addressing this, we profiled two independent pet store mice by single-cell RNAseq to define cell tropism for a spectrum of natural mouse viruses. We did observe MHV, TMEV, and MuAstV2 reads in various cell types. Despite having detectable shed viral load of MKV, we could not find any MKV reads in the intestines of these pet store mice. Importantly, we noted that the MuAstV2 viral reads clustered together with the enterocyte and immune cells clusters, particularly in the NK cell cluster. This is consistent with another study where mice lacked a functional *Il2rg*, and therefore NK cells did not shed MuAstV2 (31). Although limited conclusions can be drawn from this, we speculate that interaction between enterocytes and components of the immune system, such as the NK cells, may be required to support the infection and replication of MuAstV2 in the mouse gastrointestinal tract. Together, our findings highlight the strength of our natural transmission model in which highly unique biology of virus pathogenesis, such as MuAstV2 cell tropism, can be studied in a tractable system.

Despite kobuviruses being associated with gastroenteritis in humans and several domestic and wild mammals, they are still one of the least characterized enteric viruses. While we failed to detect MKV in the intestines by scRNAseq or ISH, we did detect MKV in the glandular stomach and gastric lymph nodes of pet store mice actively shedding MKV in their feces by ISH. However, there was no apparent pathology in any of the tissues we observed. This pattern of hybridization in the stomach was surprising because we expected that enterocytes in the small intestines would be the initial and primary target of MKV replication, similar to other enteroviruses in the *Picornaviridae* family (35, 36). Furthermore, our RNAseq data have successfully captured MKV reads in the small intestines of pet store mice infected with MKV. It is possible that our RNAseq analysis is detecting virions passing through the small and large intestines. However, in another study, we exposed deer mice to feces with high levels of MKV but did not detect any residual MKV in the small intestines after 2 or 4 days after exposure by RNA sequencing (5), suggesting that positive identification by RNAseq may not be simply due to residual virus from exposure. However, we detected some puncta in the lumen of the large intestine (Fig. 4E), suggesting that MKV virions can be detected as they are being shed in the fecal pellets. Further studies are required to further define the tropism and dynamics of MKV infection.

This cohousing model permits transmission of natural pathogens at physiologic doses. However, as we demonstrate here, pet store mice can be in different stages of infection and have variable shedding of virus species, which could impact the subsequent kinetics of transmission to cohoused mice. We defined the kinetics of virus transmission from pet store mice to cohoused B6 and IFNLAR^−/−^ mice. We showed that the rate of virus transmission to cohoused mice was different across virus species and independent experiments, highlighting the rapid and heterogeneous transmission in the dirty mouse cohousing model. We cohoused mice lacking type I and III interferon responses to evaluate how barriers imposed by the innate immune system impact transmission kinetics. Viruses transmit as early as 12 hours post-cohousing to both WT B6 and IFNLAR^−/−^ mice. We hypothesized that the lack of immune pressure in the IFNLAR^−/−^ mice would increase the rate at which the virus transmits. However, we were surprised to not observe this, suggesting that other variables, such as exposure dose, may be more critical for governing the outcome of pathogen exposure in our cohoused mouse model. Considering the coprophagic nature of mice, we suspect that some mice were continually reinoculated by fecal pellets containing a high viral load of naturally transmitted viruses. Additionally, pet store mice are often co-infected by multiple viruses with distinct kinetics, further increasing the heterogeneity in pathogen exposure in the cohousing model (Fig. 1D)(4). Altogether, results provided a valuable framework for understanding how the timing of exposure and inoculum size from the reservoir can potentially affect the outcome of transmission in natural settings.

Our study highlights significant heterogeneity in the shedding kinetics and transmission of natural mouse viruses from the pet store to the cohoused mice. Due to its heterogeneous nature, further characterization of factors such as viral dose is needed to improve the translatability of the model. We recognize that there is an inherent batch-to-batch variation between different dirty pet store mouse colonies that may carry distinct viruses that can lead to different disease manifestations and immune responses. Despite these limitations, our model provides a tractable system in which cell tropism and pathogenesis of natural mouse viruses such as MuAstV2 and MKV could be elucidated further. Characterization of the pet store mice virome will greatly improve our understanding of the important roles the viral arm of the microbiome plays in host physiology and disease.

## MATERIALS AND METHODS

### Cohousing

Pet store mice were purchased from pet stores across the Twin Cities metro area. Wild-type C57BL/6J (B6) mice were purchased from The Jackson Laboratory. B6.IL-28RA^−/−^Ifnar1^−/−^ (IFNAR^−/−^) was generated by Dr. Sergei Kotenko (37). Mice were housed in the University of Minnesota BSL-3 facility. Age-matched, non-cohoused mice served as controls and were housed in SPF facilities. For experiments done in the BSL3 facility, animals are housed in static microisolator cages. All work, including cage changes, is done in a class II, type A2 biosafety cabinet. Cage changes are done weekly with the animals removed from the dirty cage and placed into a preassembled clean cage filled with Teklad 2018 and tap water inside the biosafety cabinet.

For Fig. 1 and 3, all pet store mice were individually housed immediately upon arrival to the BSL3 facility. In Fig. 2, pet store mice from pet store A (cage 1) or pet store B (cage 2 and cage 3) were cohoused with four sets of IFNLAR^−/−^ and WT B6. At each time point (12, 24, and 48 hours post-cohousing), one set consisting of one IFNLAR^−/−^ and one WT B6 was removed from the parent cage and housed individually until endpoint to ensure the replication of transmitted viruses. Routine cage changes were done at day 4 post-initial cohousing. At day 5 post-initial cohousing, all mice from the parent cage and the time point cages were sacrificed. In Fig. 4F, two independent cohousing setups were done. For each cage, one pet store mouse was cohoused with WT B6 and IFNLAR^−/−^ for 72 hours prior to harvest. Prior to tissue harvest, all tools and work surfaces were decontaminated using 1% Virkon S to minimize pathogen cross-contamination between animals.

### Serology

Pet store mice were screened using EZ-spot cards followed by Multiplex Fluorometric ImmunoAssay (MFIA) Mouse Assessment Plus (Charles River Laboratories). The MFIA panel consists of antigens from SEND, PVM, lymphocytic choriomeningitis virus, MHV, MNV, TMEV, reovirus, epizootic diarrhea of infant mice–rotavirus, MVM, murine parvovirus 2, mouse adenovirus 1, mouse adenovirus 2, murine cytomegalovirus, polyoma virus, cilia-associated respiratory bacillus, MPUL, and *Clostridium piliforme*. Whole blood and sera were collected at the time of arrival and at sacrifice and submitted according to CRL guidelines. MuAstV2 peptide ELISA was done as previously described (12). For MKV and MnPV1 peptide ELISA, the full length of the VP1 genes was constructed according to the contigs generated from pet store mouse RNAseq data (4, 5). Peptide sequences are available in Table S1. VP1 and VP27 proteins were histidine tagged, expressed using the baculovirus expression system, and confirmed by SDS-PAGE and Western blot analysis (GenScript). The recombinant proteins were diluted in 0.1 M carbonate-bicarbonate buffer (pH 9.5) to a final concentration of 3 μg/mL upon titration and coated on 96-well or 384-well plates. Following adsorption of the antigen after incubation at 4°C overnight, the plates were washed with phosphate-buffered saline plus Tween 20 (PBST) three times and blocked with 5% skim milk at 37°C for 1.5 hours. Mouse sera were incubated on the plates at a 1:40 dilution at 37°C for 1 hour and subsequently washed three times with PBST. Bound antibody was detected with horseradish peroxidase-anti-mouse Pan IgG (Southern Biotech) antibody and detected by ABTS peroxidase substrate (SeraCare) on Synergy H1 plate reader (Biotek) at OD405.

### *In situ* hybridization

Stomach, small intestines, and large intestines were fixed in fresh 4% paraformaldehyde (pH 7.4 in PBS) for 24 hours at room temperature before washing twice in 80% ethanol. Five micrometer sections of paraffin-embedded small intestines were mounted onto Superfrost Plus slides (Thermo Fisher Scientific) 24 hours prior to RNAscope pretreatment. Slides were baked at 60°C for 1 hour prior to deparaffinization and pretreatment using the recommended protocol for RNAscope 2.5 HD Assay-RED (ACD). For target retrieval, slides were boiled in citrate target retrieval buffer at 100°C for 5 min and treated with RNAscope Protease Plus for 12 min. Slides were stained using the RNAscope Probe V-MmKoV-b187-gp1-O1-C1 specific for the MKV genome or SIVmac239 specific for SIV as a negative control, followed by RNAscope 2.5 HD-RED Detection Reagent. Slides were counterstained with filtered 50% hematoxylin and mounted in Permount (Thermo Fisher Scientific) before being scanned using the ScanScope AT2 System (Aperio Technologies). Tissues were examined by a board-certified veterinary pathologist using a post-examination method of masking (38).

### Screening for viral shedding in the fecal pellets

All pet store mice were housed individually upon arrival to the biosafety level 3 facility. On the day of arrival, fresh fecal pellets, blood, and sera were collected. RNA from fecal pellets was immediately extracted using the PowerFecal DNA/RNA kit (Qiagen) and reverse-transcribed using SuperScript II Reverse Transcription (Thermo Fisher Scientific) with oligodT_12-18_ primer. Quantitative PCR was performed with iTaq Universal SYBR Green Supermix (Bio-Rad) under the following conditions: 94°C for 2 min followed by 40 cycles of 94°C for 10 s, 58°C for 10 s, and 72°C for 30 s on Bio-Rad CFX96 Real-Time PCR Detection System. MHV (Orf1a), MuAstV2 (Orf1b), MKV (3Dpol), and MnPV1 (3Dpol) were quantified using standard curves from 10-fold dilutions of geneblock standards (Integrated DNA Technologies). Primers sequences can be found in Table S1.

### RNA sequencing and analysis

Two RNAseq experiments were analyzed. In the first experiment (Fig. 2B), small intestines, liver, and lung were collected. In the second (Fig. 4F) stomach, small intestines and large intestines were collected. Tissues were homogenized in GentleMacs M tubes (Miltenyi Biotec) in Buffer RLT Plus (Qiagen) supplemented with 2-mercaptoethanol (10 µL/1 mL) and Reagent DX (0.5% vol/vol, Qiagen). RNA was extracted using the AllPrep DNA/RNA Mini kit (Qiagen). PolyA-enriched cDNA libraries were prepared using the Kapa Hyper Stranded mRNA library prep kit (Roche). Sequencing was performed using NovaSeq 6000 with 150 bp paired-end reads. RNAseq data were generated by mapping reads to the *Mus musculus* genome using STAR (v.2.7.1a) (39). For each experiment, unmapped reads from all animals and tissue types were concatenated and assembled using *de novo* assembly with Trinity (v.2.12) (40). Contigs below 500 bp were removed from further analysis, and the remaining contigs were assigned for their taxonomic lineages using BLASTn (41). Estimated transcript abundances (transcripts per million) were obtained by mapping reads to an index of the assembled, filtered contigs using Salmon (v.1.4) (42). Transcript abundances were summarized at either species or family level and graphed with ggplot2 (43) in R (R Core Team, 2018, R: A Language and Environment for Statistical Computing [v.3.5.2]; R Foundation for Statistical Computing, Vienna, Austria). Downstream analyses only included data from small intestine for the experiment in Fig. 2 and from stomach tissues for the experiment in Fig. 4 to focus on viruses with gastrointestinal tropism.

### Single-cell RNA sequencing and analysis

Single-cell suspensions from pooled mouse small and large intestines were isolated as previously described (30) with minor modifications. Briefly, tissues were rinsed, and lumen was flushed with cold PBS before being opened longitudinally and sliced into small fragments. Tissues were incubated in 20 mM EDTA-PBS on ice for 90 min while shaking every 30 min. At every 30 min increment, the tissues were shaken vigorously by hand, and the supernatant was collected into a new conical tube. The tissue was incubated in fresh EDTA-PBS, and a new fraction was collected every 30 min. The final fraction was washed twice in PBS, centrifuged at 300 × *g* for 3 min, and dissociated with 2 mL TrypLE express (Invitrogen) for 1 min at 37°C. Dissociated tissues were passed through a 40 µm filter on ice before centrifuging at 300 × *g* for 3 min and resuspending in 5 mL of 10% bovine serum albumin in PBS. Ten thousand cells were loaded onto the Chromium Controller (10x Genomics) to partition single cells into gel beads. Single-cell transcriptomic libraries were generated using the Chromium Next GEM Single Cell 3′ Kit (v.3.1, 10x Genomics) according to the manufacturer’s instructions. Libraries were sequenced on the NovaSeq X Plus (Illumina), generating 25 B reads per sample.

Cell Ranger (v.9.0.0, 10x Genomics) was used to first process the gene expression data using a customized version of the mm10 reference that also included the complete genomes of MKV (accession number PV544232), MnPV1 (accession number PV544233), MHV (accession number PV544234), MuAstV2 (accession number PV544235), and TMEV (accession number PV544236). Mapped filtered feature barcode matrices were analyzed in Seurat (v.5.1.0) (44). Samples were normalized by the median number of mapped reads per identified cell. Data were first filtered by excluding the cells that exhibited extremes in the distributions of the number of genes expressed (<250 and >5,000), the number of mRNA molecules (>50,000), and the percent expression of mitochondrial genes (>50%). The cutoff was chosen to prevent the elimination of cells that undergo anoikis. Conserved feature markers per cluster were identified and annotated using a combination between ScType (45), the mouse gastrointestinal marker database from CellMarker (v.2.0), and established markers from literature (30). We manually assigned the “tuft cell” cluster to replace the “cancer stem cell” cluster annotated by ScType due to the high expression of Dclk1, a canonical marker for tuft cells. Goblet cells, a cell type that gradually increases in proportion from the duodenum to the distal colon, did not cluster separately; rather, they are clustered in the deep crypt secretory cell cluster. Expression levels of Dclk1 for tuft cells and Muc2 for goblet cells can be found in Fig. S3.

## Data Availability

All sequencing data from this study were deposited and are publicly available as FASTQ files in the NCBI Sequence Read Archive under BioProject ID PRJNA1251036. The code used to analyze these data is publicly available at https://github.com/langloislab/jvi_putri_2025.

## SUPPLEMENTAL MATERIAL

Supplemental figures jvi.00187-25-s0001.pdf Figures S1 to S3.

Table S1 jvi.00187-25-s0002.xlsx Primer sequences used for quantifying fecal viral shedding by qRT-PCR in Fig. 1A.

Table S2 jvi.00187-25-s0003.xlsx Peptide sequences used as antigens for MuAstV2, MKV, and MnPV1 peptide ELISA.

ASM does not own the copyrights to Supplemental Material that may be linked to, or accessed through, an article. The authors have granted ASM a non-exclusive, world-wide license to publish the Supplemental Material files. Please contact the corresponding author directly for reuse.
