# Supplementary material for: Naturally transmitted mouse viruses highlight the heterogeneity of virus transmission dynamics in the dirty mouse model
Source: J Virol. 2025 May 28;99(6):e00187-25. doi: 10.1128/jvi.00187-25 (PMC12172463; doi:10.1128/jvi.00187-25)
Supplement: Supplemental figures — Figures S1 to S3. [file jvi.00187-25-s0001.pdf]

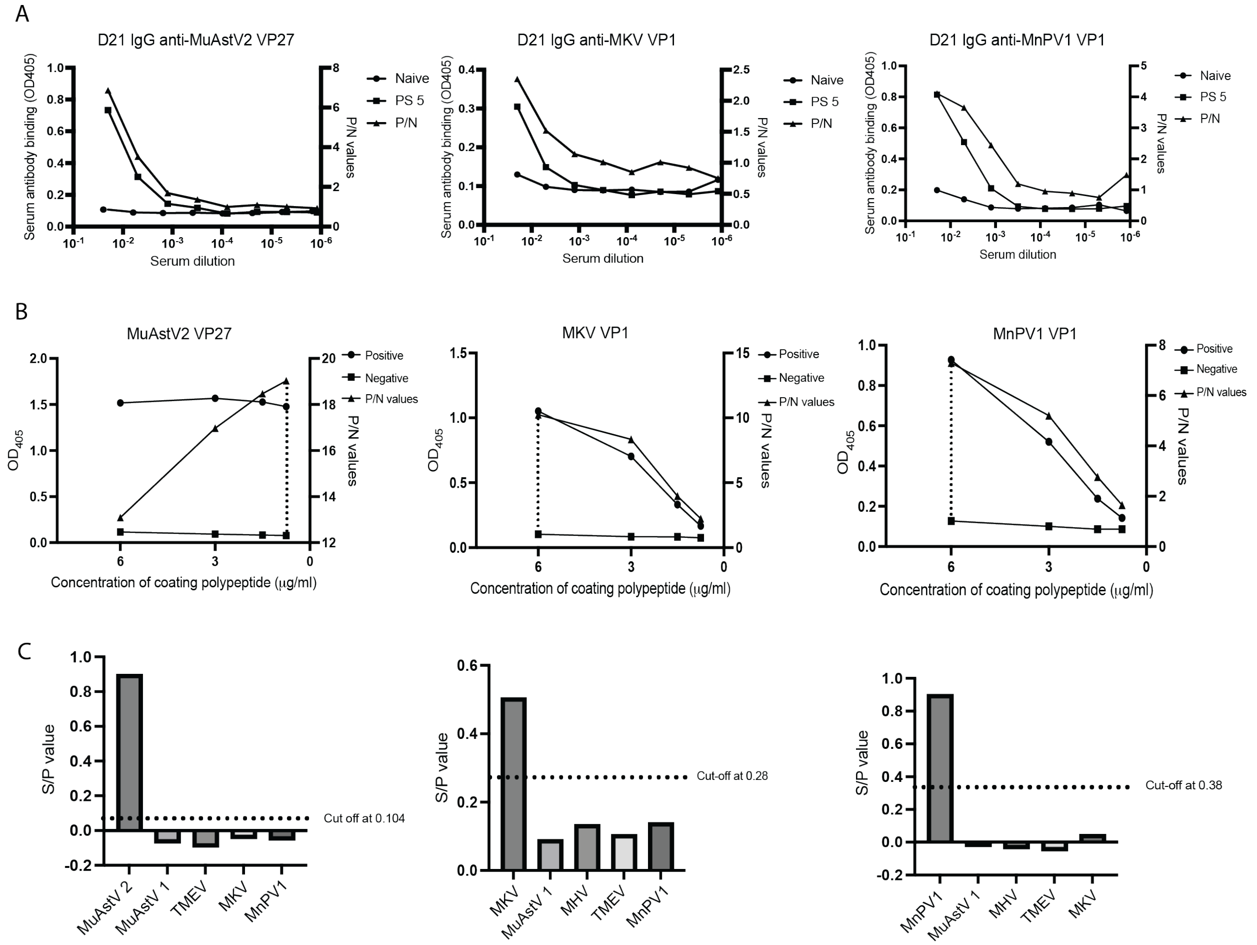

Supplemental Figure 1. Optimization of peptide ELISA for MuAstV2, MKV, and MnPV1. Titration assays were performed to optimize the working concentration of the mouse serum dilution (A) and coating polypeptide (B). The vertical dotted lines indicate conditions selected for using in the downstream assays. P/N values represent the ratios of mean OD values obtained for positive sera compared to those obtained for negative sera. (C) Specificity of peptide ELISA was determined by calculating the cutoff value from animal that is seropositive for one virus and seronegative for the rest. S/P value was determined as ((sample OD_{450} value-negative standard OD_{450} value) / (positive standard OD_{450} value-negative standard OD_{450} value))(1). The cutoff value was determined as mean + 3*standard deviation, as previously described(2).

Top Public Peptide Recognitions

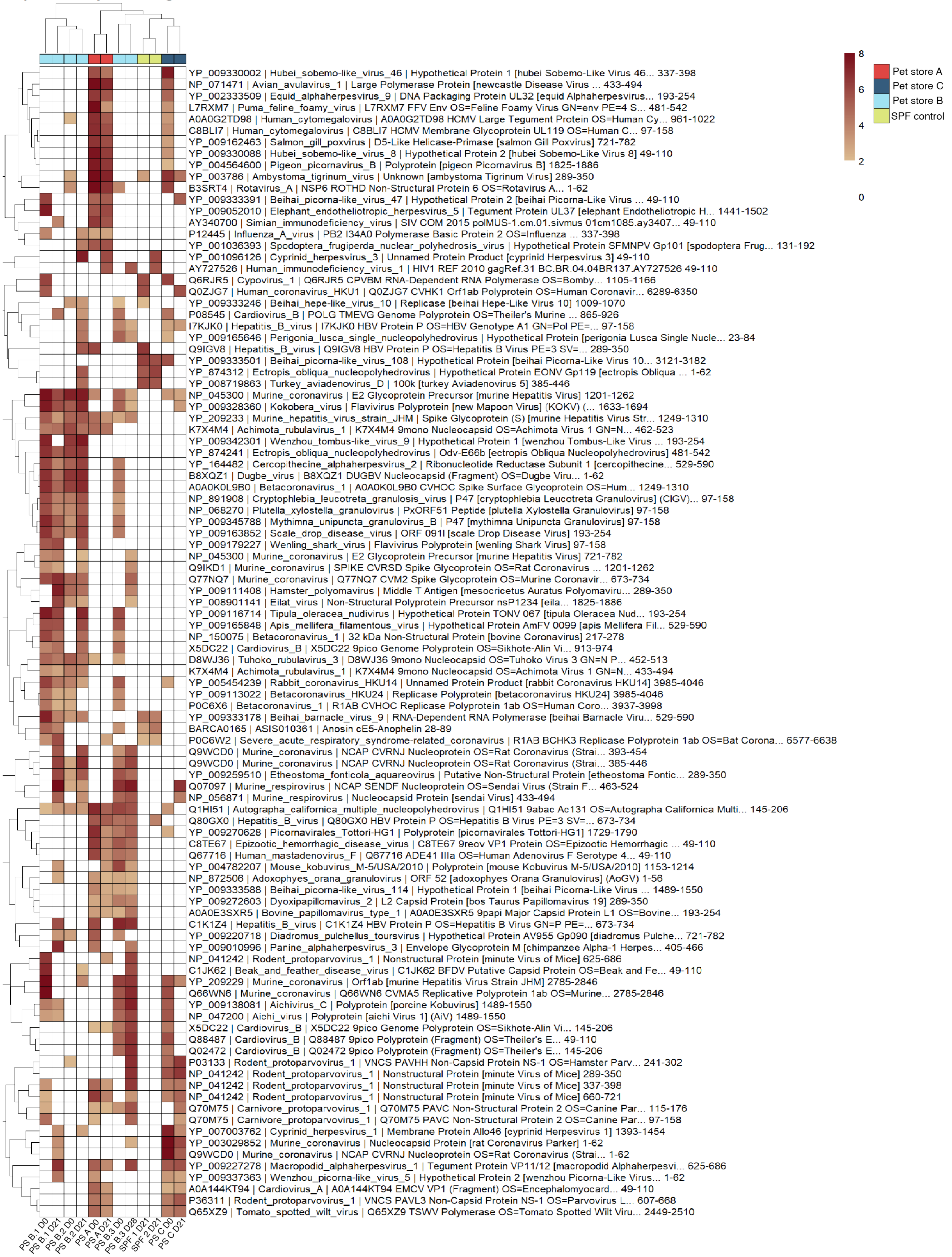

Supplemental Figure 2. Top public clustered heatmap of VirScan analysis. Unbiased summary heatmap of the top public reactive peptides from the top_public_peptides.csv output rows (https://doi.org/10.6084/m9.figshare.28835711.v1). Heatmap was created in R using the pheatmap package with default row and column clustering settings. Scale is Log2 fold change. Euclidean distance was used as the similarity measure and clustering samples.

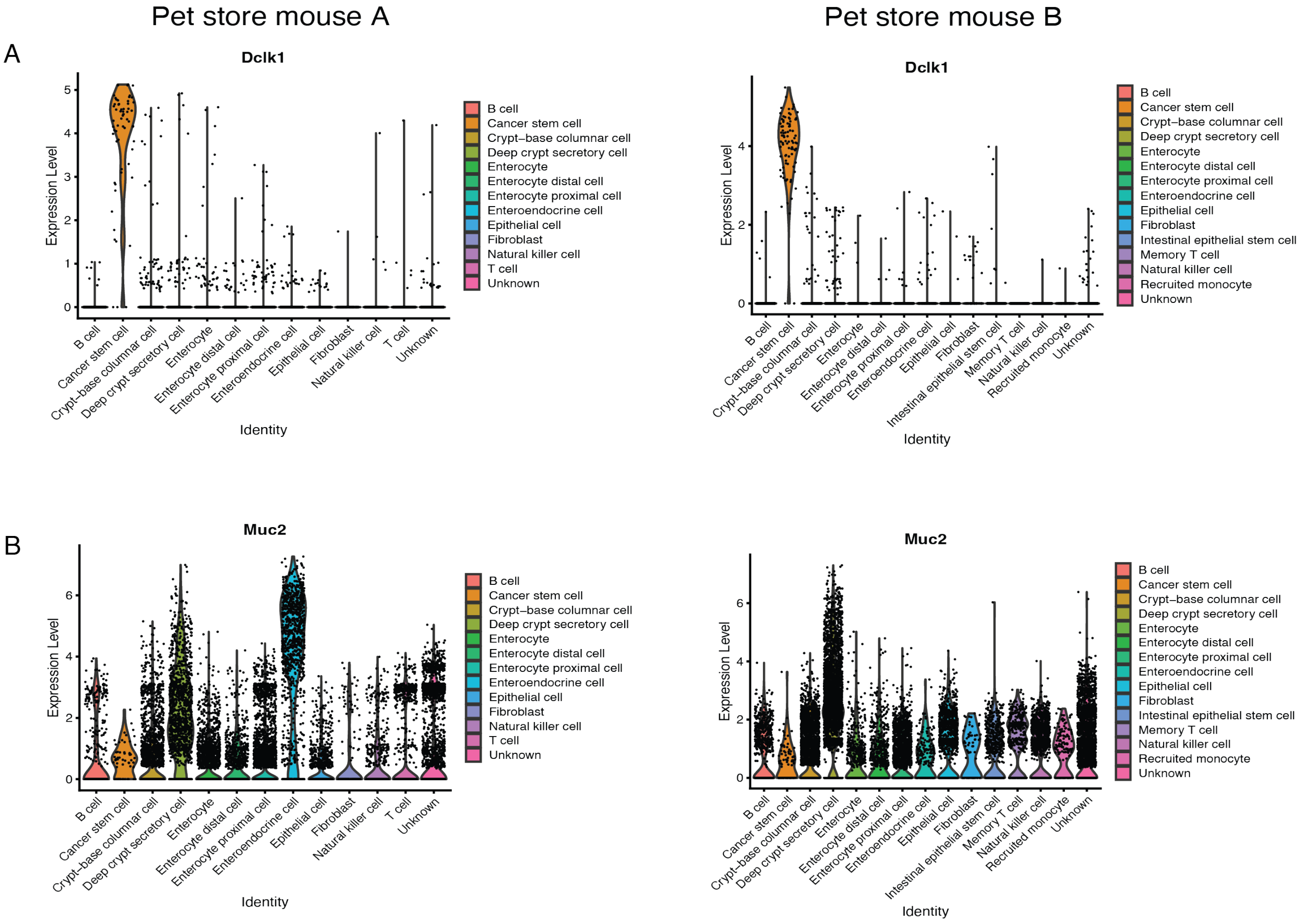

Supplemental Figure 3. Gene expressions of Dclk1 and Muc2 markers. (A) Dclk1 expression is the highest in the cancer stem cell cluster for both pet store mouse A and B. (B, *left*) In pet store mouse A, Muc2 expression is high in the deep crypt secretory cell and enteroendocrine cell clusters whereas in pet store mouse B, Muc2 expression is the highest in the deep crypt secretory cell cluster (*right*).

Table S1

Primer sequences used for quantifying fecal viral shedding by qRT-PCR in Figure 1A.

Table S2

Peptide sequences used as antigens for MuAstV2, MKV, and MnPV1 peptide ELISA.

References

1. Jung A, Rautenschlein S. 2020. Development of an in-house ELISA for detection of antibodies against Enterococcus cecorum in Pekin ducks. Avian Pathol 49:355-360.
2. Ren D, Zhang X, Zhang W, Lian M, Meng X, Li T, Xie Q, Shao H, Wan Z, Qin A, Gao W, Ye J. 2023. A peptide-based ELISA for detection of antibodies against novel goose astrovirus type 1. J Virol Methods 312:114646.
